# Blood N1-methyladenosine (m^1^A) RNA modification and outcome after cardiac arrest

**DOI:** 10.1186/s13054-026-05906-0

**Published:** 2026-02-25

**Authors:** Victoria Stopa, Miron Sopic, François Bernardin, Nathalie M. Legrave, Lu Zhang, Pascal Stammet, Yvan Devaux

**Affiliations:** 1 Cardiovascular Research Unit, Department of Precision Health, Institute of Health, 1A-B rue Edison, Strassen, L-1445 Luxembourg; 2 Department of Medical Biochemistry, Faculty of Pharmacy, University of Belgrade, Belgrade, Serbia; 3 Metabolomics Platform, Department of Cancer Research, Institute of Health, 1A-B rue Edison, Strassen, L-1445 Luxembourg; 4 Bioinformatics and AI Unit, Department of Medical Informatics, Luxembourg institute of Health, Strassen, Luxembourg; 5 Department of Anesthesia and Intensive Care Medicine, Centre Hospitalier de Luxembourg, Luxembourg, Luxembourg; 6 Department of Life Sciences and Medicine, Faculty of Science, Technology and Medicine, University of Luxembourg, Esch-sur-Alzette, Luxembourg

**Keywords:** RNA methylation, Cardiac arrest – prognostication, Biomarkers

## Abstract

**Background:**

Cardiac arrest (CA) is a leading cause of mortality and neurological disability. Prediction of post-CA outcomes is challenging. Epitranscriptomic (RNA) modifications are emerging as potential biomarkers due to their regulatory roles in RNA metabolism and disease progression. However, their relevance in CA remains unexplored.

**Objective:**

This study aimed to investigate the association between N1-methyladenosine (m¹A) RNA modification and outcome after CA.

**Methods:**

Total RNA was extracted from whole blood samples of 211 patients collected 48 h after return of spontaneous circulation (ROSC). M¹A and adenosine (A) blood levels were quantified using liquid chromatography coupled to mass spectrometry (LC-MS), and the ratio m¹A/A was calculated. Neurological outcome assessed using the cerebral performance category (CPC) score and survival at 6 months were used as end-points.

**Results:**

Patients with moderate to severe neurological outcome or death within 6 months after CA (CPC 2–5) exhibited elevated m¹A/A ratio compared to survivors without neurological sequelae (CPC 1) (*p* = 0.03). In multivariable logistic regression, higher m^1^A/A levels were associated with an increased risk of moderate to severe neurological outcome or death at 6 months compared to survivors without neurological sequelae (odds ratio [95% confidence interval] 1.50 [1.04–2.19]), after adjustment for age, time between CA and return of spontaneous circulation, lactate and neuron-specific enolase levels. In Kaplan-Meier survival analysis, patients with elevated m¹A/A levels showed a lower probability of survival at 6 months (*p* = 0.003).

**Conclusion:**

This study provides the first evidence that m^1^A RNA methylation, reflected by the m^1^A/A ratio, is associated with neurological outcome and death at 6 months after CA. Although these findings require validation, they raise the possibility that m¹A RNA methylation could help to improve prognostication after CA.

## Introduction

Cardiac arrest (CA) is the sudden cessation of cardiac activity and remains a major global public health challenge, accounting for approximately 20% of all adult deaths in the USA and Western Europe [1]. While advancements in advanced life support, post-resuscitation care, and public awareness campaigns have improved CA management, survival rates remain low [2], with more than 80% of patients not surviving. Among survivors, approximately 80% achieve a favorable neurological outcome and recovery, while the remaining proportion experience varying degrees of neurological impairment due to hypoxic-ischemic brain injury [3]. This injury occurs in two phases: first, the initial ischemic phase due to the cessation of cerebral blood flow when the heart stops, and second, the reperfusion injury, which results from the restoration of circulation and oxygenation which triggers a cascade of pathological mechanisms that exacerbate neuronal damage [3, 4].

Predicting neurological outcome after CA remains a significant clinical challenge and relies on clinical examination, outcome assessment scores such as the Cerebral Performance Category (CPC) scale, Glasgow Coma Scale (GCS), modified Rankin Scale (mRS), and neurophysiological measures as electroencephalography (EEG), and somatosensory evoked potentials (SSEP) and neuroimaging [5]. In addition, biomarker-based approaches, including the well-established neuron-specific enolase (NSE) [6], and more emerging promising biomarkers such as the neurofilament light (NfL) [7], tau protein (Tau), and ubiquitin carboxy-terminal hydrolase L1 (UCH-L1) [8], have been explored. However, these markers still have some limitations in accuracy and specificity, sometimes leading to misclassification. Although these biomarkers directly reflect neuronal or glial injury, they present important limitations including extracerebral sources, susceptibility to confounding factors, and variability in optimal timing and thresholds. While biomarkers such as Nfl are now close to clinical implementation [7], others remain at the research stage. This highlights the need for novel prognostic biomarkers and the need to gain deeper insights into the underlying regulatory mechanisms of post-CA injury. To address the limitations of currently available markers, multicomponent approaches integrating various omics technologies, alongside clinical assessments, neuroimaging, electrophysiological techniques, and classical biomarker measurements, are being investigated to improve prognostication [9]. Among these emerging approaches, epitranscriptomics, the study of RNA modifications, holds promise for uncovering novel biomarkers that could enhance post-CA outcome prediction but also for revealing molecular mechanisms underlying post-CA injury and recovery. RNA modifications play essential roles in regulating gene expression, RNA stability, splicing, translation efficiency, and degradation [10, 11], thereby influencing critical biological processes such as neuronal survival, stress response, inflammation, and neurodegeneration.

More than 170 distinct RNA modifications have been identified to date [11] N6-methyladenosine (m6A) is the most extensively studied, with well-established roles in RNA metabolism, translation and pathophysiology. However, the biological and clinical relevance of other RNA modifications remains less characterized and their potential as biomarkers is still poorly characterized.

Among these less-well studied modifications, N1-methyladenosine (m¹A), characterized by the addition of a methyl group to the N1 position of adenosine, has emerged as a functionally relevant RNA modification involved in the regulation of RNA structure, RNA–protein interactions, and mitochondrial RNA metabolism [12]. M¹A is dynamically regulated by a dedicated enzymatic machinery. It is installed by writers such as TRMT61B, which modifies both transfer RNAs (tRNAs) and mitochondrial mRNAs/ribosomal RNAs, and TRMT61C, which targets tRNAs and mitochondrial mRNAs.The modification is reversible, with erasers including ALKBH1, ALKBH3, ALKBH7, and FTO mediating its removal. Additionally, members of the YTHDF protein family function as readers, recognizing and interpreting m¹A to influence RNA metabolism [12]. Although m¹A is generally present at relatively low abundance, its functional significance in RNA biology and diseases is increasingly being explored. Although research on m¹A in cardiovascular and neurological diseases remains limited, its involvement in RNA structural regulation, mitochondrial RNA function, and cellular stress responses suggests a potential role in disease pathophysiology. Emerging data also suggest that RNA methylation is altered in cerebral ischemia, with changes in m¹A linked to neuronal injury and inflammatory responses [13].

These findings highlight a potential link between epitranscriptomic regulation and hypoxic–ischemic brain injury. Therefore, in the present study, we examined whether circulating m¹A levels measured 48 h after ROSC were associated with neurological outcome and survival after CA.

## Materials and methods

### Patients and study design

In this prospective, monocentric observational study, 211 adult patients from the Luxembourgish monocenter “North Pole study” cohort for whom blood samples for RNA study were available, have been enrolled [14]. These patients experienced CA between 2008 and 2020. Participants were admitted to the intensive care unit following either out-of-hospital or in-hospital CA. The study was approved by the Luxembourg National Ethics Committee (CNER N°200803/05), and informed consent was obtained from either the patients or their relatives, in accordance with the Declaration of Helsinki and Luxembourg’s legislative requirements.

Whole blood samples were collected in PAXgene Blood RNA tubes (PreAnalytiX, Cat. N° 762165), 48 h after ROSC. Total RNA was extracted using PAXgene Blood RNA kit (Qiagen), following the manufacturer’s recommendations. RNA concentration was determined using a Nanodrop 1000 spectrophotometer (Thermo Fisher Scientific).

Demographic, clinical, resuscitation, and biochemical data were prospectively collected alongside sample collection. Covariates for statistical analysis and multivariable modeling were determined based on their established association with the neurological outcome and availability of complete data across the cohort. Blood Lactate, NSE and CRP were measured at 48 h after ROSC, as part of clinical routine. For out-of-hospital CA, time from cardiac arrest to ROSC was defined as the interval between the estimated time of collapse as reported by witnesses or emergency medical services or the time of the emergency call and the time of sustained ROSC documented by the emergency medical team. No patients in the present cohort received Extra Corporeal Membrane Oxygenation (ECMO). However, the majority of patients underwent post-cardiac arrest cardiac catheterization.

### Study end-point

After a 6-month follow-up, the neurological outcome was assessed using the CPC score for all the 211 patients studied. This neurological outcome served as the primary endpoint of the study. CPC 1 indicated surviving patients without neurological sequelae, while CPC 2–5 represented patients with increasing severity of neurological sequelae, ranging from moderate to severe, with CPC 5 indicating death. The six-month neurological outcome was assessed using the CPC score, obtained through standardized telephone interviews conducted 6 months after cardiac arrest or by medical chart review (from cardiologists or neurologist visits). Only patients with the CPC score obtained at 6 months were included in the present study, no patients were lost to follow-up for this outcome. Information on cause of death was available for a subset of patients, and were predominantly neurological, including post-anoxic encephalopathy, cerebral edema, brain death, or refractory status epilepticus. Other reported causes included cardiovascular collapse, multiple organ failure, and, less frequently, septic shock. Neurological outcome was assessed using the 6-month CPC, reflecting global functional outcome rather than specific mechanisms of death.

Several RNA modifications were quantified in this study, including N⁶-methyladenosine (m⁶A), N⁶−2′-O-methyladenosine (m⁶Am), 2′-O-methyladenosine (Am), β-pseudouridine (Ψ), and m¹A. Among these, only m¹A and the m¹A/A ratio showed a significant association with the primary endpoint of neurological outcome at 6 months. The other RNA modifications were tested in the same analytical framework but were not significantly associated with the outcome.

### RNA sample preparation

RNA sample preparation was performed according to a protocol described previously [15]. Briefly, 500 ng of total RNA was digested with Nuclease P1, 100U/ul (NEB#M0660S) at 37 °C for 3 h to cleave the RNA into nucleoside monophosphates. Following this step, the samples were treated with calf intestinal phosphatase (NEB#M0525S) at 37 °C for 2 h followed by heat inactivation for 2 min at 80 °C to dephosphorylate the nucleotides. The resulting nucleosides were then used for downstream LC-MS measurements as follows: 25 µL aliquot was mixed with 100 µL methanol containing 0.065 nmol d_3_-m^6^A as internal standard. The mixture was subsequently evaporated and resuspended in 40 µL water containing 20 mM ammonium formate.

### Liquid-chromatography coupled to mass spectrometry (LC-MS) measurement

Metabolite analysis was performed using a Vanquish^®^ Horizon UHPLC system (Thermo Scientific) coupled to an Orbitrap^®^ Exploris480^®^ mass spectrometer (Thermo Scientific). Chromatography was carried out with a Phenomenex Kinetex 2.6 μm EVO C18 100Å, LC column 150 × 2.1 mm protected by a SecurityGuard ULTRA cartridges for EVO-C18 UHPLC.

The temperature of the column was kept at 40 °C and the flow rate was set to 0.3 ml/min. The mobile phases consisted of 20 mmol/L ammonium formate in water, pH 3.5 (eluate A) and methanol (eluate B), loading and gradient occurred at 0.3mL/min flow rate, loading step lasted 6 min with 2% eluate B. Then started a linear gradient ranging from 2% eluate B to 95% eluate B in 3 min. After 19 min started the regeneration step with 95% eluate B. From 12.5 min until 21 min, the column is re-equilibrated with 2% eluate B at a flow rate of 0.3mL/min. 5 µL of sample were injected into the instrument. The MS experiment was performed using electrospray ionization with polarity switching enabled. Source parameters were as follows: sheath gas flow rate, 40; aux gas flow rate, 8; sweep gas flow rate, 1; spray voltage, 3,5 kV (+)/2,5 kV (-); capillary temperature, 350 °C; S-lense RF level, 50; aux gas heater temperature, 150 °C. The Orbitrap mass analyzer was operated at a resolving power of 120,000 (at 200 m/z) in full-scan mode (scan range: m/z 75–800; automatic gain control target set to 1e6 charges with an intensity threshold of 5e3, MS2 was perform at Orbitrap resolution of 15000).

### Analysis of LC-MS data

Data from LC-MS measurements were acquired with the Thermo Xcalibur software (Version Version 4.7.69.37) and analyzed using TraceFinder (Version 5.1). Absolute concentrations of m¹A and adenosine (A) were determined in ng/mL of RNA extract using external calibration curves from pure standards spiked in aliquots of pooled samples. To minimize inter-individual variability in total A content, results were expressed as the m¹A/A ratio, calculated as the concentration of m¹A divided by that of A. This ratio reflects the relative methylation level rather than an absolute quantity. Quantification was based on chromatographic signal intensity.

### Statistical analysis

Statistical analysis was performed using both Sigma Plot 16.0 and R Studio. Mann-Withney U test was used to compare numeric variables between two groups. Chi-square test was used to compare categorical characteristics of patients according to their neurological outcome. Missing clinical data were imputed using the R package MissForest for clinical covariates only, and no imputation was performed for the m^1^A/A ratio or the neurological outcome. Data were log transformed and scaled. Univariate and multivariable logistic regression were performed to assess the association of m¹A with 6-month neurological outcome.

The quality of the model when adding the m¹A/A ratio to a baseline clinical model was evaluated using the Akaike Information Criterion (AIC) and the likelihood ratio test.

Reclassification analyses were performed using the net reclassification improvement (NRI) and the integrated discrimination improvement (IDI), as previously described [16]. Kaplan-Meier survival curves and Cox proportional hazards models were used to estimate the association between m¹A and 6-month survival. A p-value < 0.05 was considered statistically significant.

## Results

### Patient demographics and clinical data according to the neurological outcome

We first used 6-month neurological outcome as primary end-point. As shown in Table 1, patients with good neurological outcome (i.e., who survived without neurological sequelae; CPC 1) had a median age of 56.5 years as compared to patients with moderate to severe neurological outcome or death (CPC 2–5) that were older (median 65 years). Sex distribution and body mass index were comparable between groups. The prevalence of comorbidities such as smoking, coronary artery disease, heart failure, hypertension, and diabetes mellitus did not differ significantly between groups.

Variables related to resuscitation and biochemical injury showed significant differences. Patients with neurological sequelae or death were less likely to receive bystander CPR (57% vs. 77%, *p* = 0.005) and had a significant longer time from CA to return of spontaneous circulation (ROSC) (*p* = 0.04). They also had significant higher lactate concentrations (*p* = 0.019), higher neuron-specific enolase (NSE) levels at 48 h (*p* < 0.001), and increased C-reactive protein (CRP) levels at 48 h (*p* = 0.035).

**Table 1:** Demographic and clinical data of the 211 patients of the study cohort according to 6-month neurological outcome

Characteristics	Neurological outcome		*p*-value
	*CPC 1 (n = 70)*	*CPC 2–5 (n = 141)*	
*Age (years)*	56.5 (18–83)	65 (24–87)	0.11
*Sex*			
* Male*	58 (83%)	107 (76%)	0.25
* Female*	12 (17%)	34 (24%)	
*BMI*	25.9 (17.05–39.18)	27 (15.15–55.53)	0.221
*Comorbidities*			
* Smoker*	32 (46%)	58 (41%)	0.52
* Coronary diseases*	20 (29%)	47 (33%)	0.48
* Heart failure*	23 (33%)	52 (37%)	0.57
* Hypertension*	26 (37%)	66 (47%)	0.18
* Diabetes mellitus*	9 (13%)	18 (13%)	0.99
*Bystander CPR*	54 (77%)	81 (57%)	**0.005**
*Time between CA and ROSC (min)*	21.5 (5–90)	30 (5–104)	**0.04**
*Lactate (mmol/l)*	2.65 (0.68–12.08)	3.96 (0.46–19.26)	**0.019**
*NSE 48 h after ROSC (ng/ml)*	18.35 (7.90–94.40)	75 (7.20–875)	**< 0.001**
*CRP 48 h after ROSC (mg/l)*	118 (30–330)	140 (16.60–440)	**0.035**

Patients with neurological sequelae or death (CPC 2–5) were less likely to receive bystander CPR, and had longer time to ROSC, higher lactate levels, elevated NSE levels, and higher CRP levels compared with patients without neurological impairment (CPC 1). Categorical variables are presented as number (proportion) and continuous variables are indicated as median (range). (BMI) Body Mass Index, (CPR) Cardiopulmonary resuscitation, (ROSC) Return of spontaneous circulation, (CA) Cardiac arrest, (NSE) Neuron specific enolase, (CRP) C-reactive protein. Bold values indicate statistical significance (p < 0.05)

### Patient demographics and clinical data according to survival

We then used 6-month survival as secondary end-point. As shown in Table 2, non-survivors (CPC 5) were significantly older than survivors (CPC 1–4) and less likely to receive bystander CPR (*p* = 0.012). They also showed a longer time from CA to ROSC (*p* = 0.01) and higher lactate and NSE concentrations at 48 h (*p* = 0.004 and *p* < 0.001, respectively). However, sex distribution, BMI, comorbidities and CRP levels did not differ significantly between groups.

**Table 2:** Demographic and clinical data of the 211 patients of the study cohort according to 6-month survival

Characteristics	Survival		*p*-value
	*CPC1-4 (n = 113)*	*CPC5 (n = 98)*	
*Age (years)*	58.5 (18–87)	65 (24–87)	**0.021**
*Sex*			
*Male*	92 (81%)	73 (74%)	0.22
*Female*	21 (19%)	25 (26%)	
*BMI*	26.36 (15.15–55.53)	27.02 (15.15–52.85)	0.058
*Comorbidities*			
*Smoker*	50 (44%)	40 (41%)	0.62
*Coronary diseases*	31 (27%)	36 (37%)	0.15
*Heart failure*	37 (33%)	38 (39%)	0.36
*Hypertension*	46 (41%)	46 (47%)	0.36
*Diabetes mellitus*	10 (9%)	17 (17%)	0.065
*Bystander CPR*	81 (72%)	54 (55%)	**0.012**
*Time between CA and ROSC (min)*	24.50 (5–90)	30 (5–104)	**0.01**
*Lactate (mmol/l)*	3.27 (0.46–13.33)	4.15 (0.46–19.26)	**0.004**
*NSE 48 h after ROSC (ng/ml)*	25.70 (7.20–768)	84.95 (7.20–875)	**< 0.001**
*CRP 48 h after ROSC (mg/l)*	130.50 (16.60–440)	143.50 (16.60–440)	0.124

Patients who died were older than survivors, less frequently received bystander CPR, and had longer time to ROSC. They also showed higher lactate levels and elevated NSE concentrations at 48 h post-ROSC. Categorical variables are presented as number (proportion) and continuous variables are indicated as median (range). (BMI) Body Mass Index, (CPR) Cardiopulmonary resuscitation, (ROSC) Return of spontaneous circulation, (CA) Cardiac arrest, (NSE) Neuron specific enolase, (CRP) C-reactive protein. Bold values indicate statistical significance (p < 0.05)

### Assessment of m^1^A levels and association with 6-month neurological outcome and survival

**Fig. 1:**
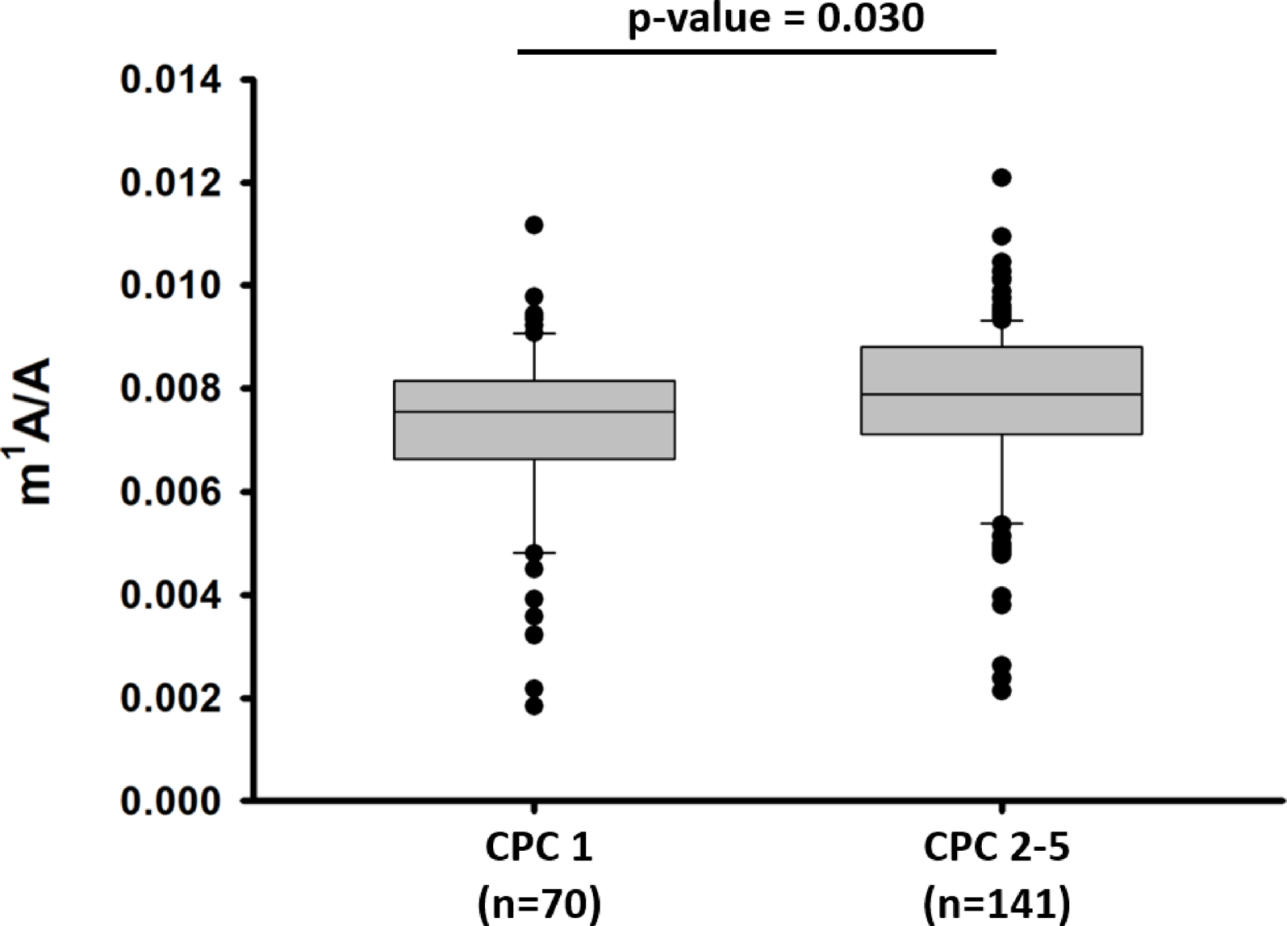
m¹A/A ratio according to 6-month neurological outcome. Boxplots show the m¹A/A ratio in patients with good neurological outcome (CPC 1, *n* = 70) and patients with moderate to severe neurological outcome or death (CPC 2–5, *n* = 141). The m¹A/A ratio was significantly increased in the CPC 2–5 group compared to the CPC 1 group (Mann–Whitney U test, *p* = 0.030)

Total RNA extracted from whole blood samples of 211 patients was analysed by LC-MS for nucleoside content. We obtained the levels of m^1^A and A, and calculated the ratio m^1^A/A to account for inter-individual variability of A content. The ratio m¹A/A was compared between patients with good neurological outcome (CPC 1, *n* = 70) and patients with moderate to severe neurological outcome or who died (CPC 2–5, *n* = 141). We observed that m¹A/A ratio was increased (*p* = 0.030) in patients with moderate to severe neurological outcome or death (CPC 2–5) as compared to patients with good neurological outcome (CPC 1) (Fig. 1). m¹A/A ratios across individual CPC categories are shown in Supplementary Fig. 1, illustrating a modest increase with worsening outcome but substantial overlap between groups.

### Assessment of the potential of m¹A/A ratio to predict neurological outcome

**Fig. 2:**
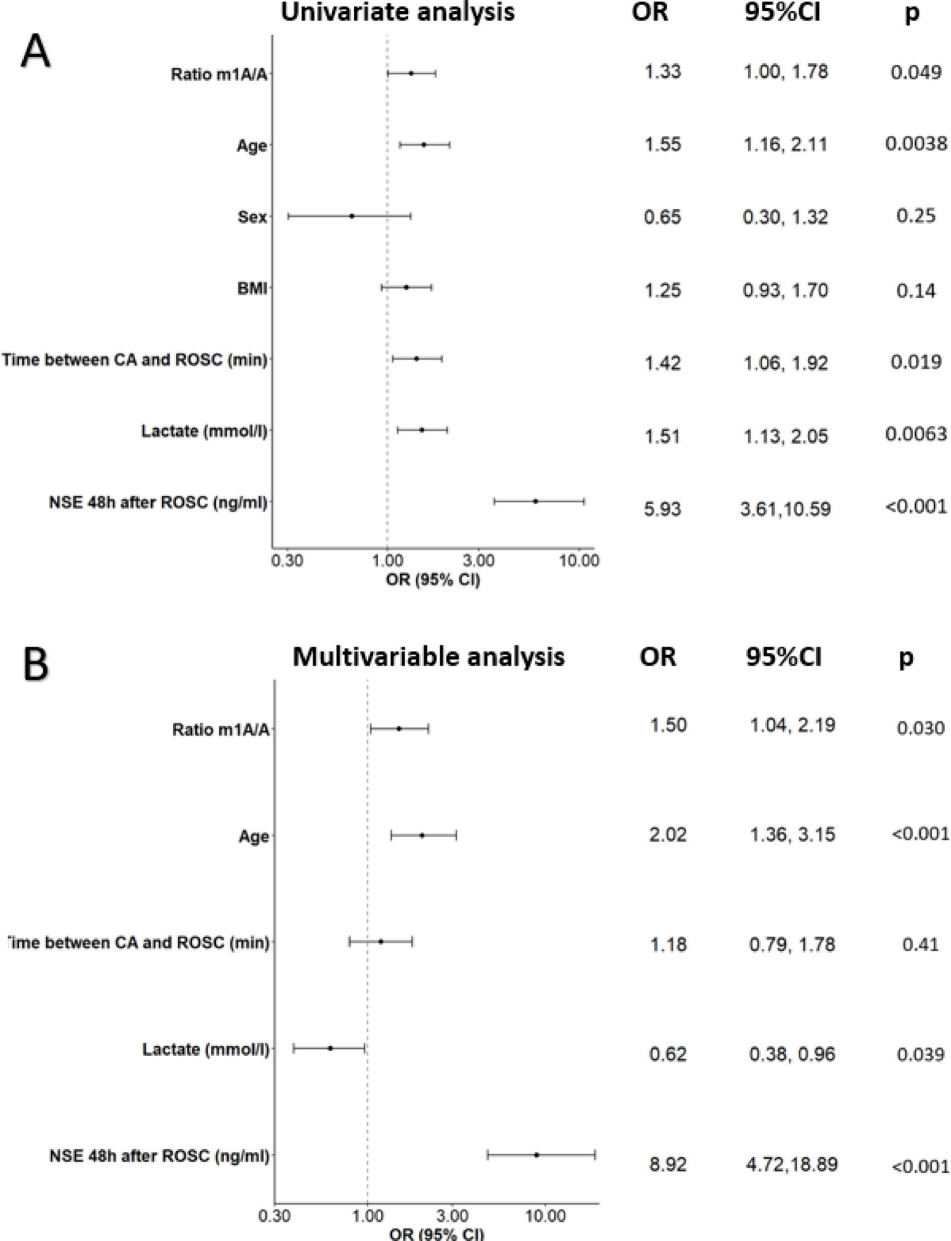
Univariate and multivariable logistic regression analysis assessing the association between m^1^A/A ratio, clinical variables and moderate to severe neurological outcome or death (CPC2-5) at 6 months in 211 CA patients. Forest plots with odds ratios (OR) and 95% confidence intervals (CI) are shown. (**A**) In univariate analysis, higher m¹A/A ratio, age, time between CA and ROSC, lactate, and NSE measured at 48 h were significantly associated with poorer neurological outcome. NSE showed the strongest association. (**B**) In multivariable analysis, after adjustment for clinical covariates, m¹A/A ratio remained independently associated with neurological outcome, together with age and NSE. NSE remained the strongest independent predictor

Univariate logistic regression analysis was first performed to assess the association between m¹A/A, clinical variables and neurological outcome or death (CPC 2–5). We observed that m¹A/A ratio was associated with moderate to severe neurological outcome or death (odds ratio (OR): 1.33, 95% confidence interval (CI): 1.00–1.78, *p* = 0.049)) (Fig. 2A). In addition, significant associations were observed for age (OR 1.55, 95% CI 1.16–2.10, *p* = 0.004), time between cardiac arrest (CA) and return of spontaneous circulation (ROSC) (OR 1.42, 95% CI 1.06–1.91, *p* = 0.019), lactate levels (OR 1.51, 95% CI 1.12–2.05, *p* = 0.006), and NSE at 48 h after ROSC (OR 5.93, 95% CI 3.61–10.59, *p* < 0.001). No significant association was identified for sex or BMI. After Bonferroni correction for multiple univariate comparisons across the five RNA modification ratios tested, the association between the m1A/A ratio and poor neurological outcome was no longer statistically significant (adjusted *p* = 0.245).

We then conducted multivariable logistic regression analysis in which variables that showed significant associations with neurological outcome in the univariate analysis were included in the model (m¹A/A ratio, age, time between CA and ROSC, lactate, and NSE at 48 h after ROSC). We observed a significant association between the m¹A/A ratio and neurological outcome or death (CPC 2–5) (Fig. 2B). Patients with elevated m¹A/A ratio had a 50% higher risk of moderate to severe neurological outcome or death as compared to patients with lower m¹A/A ratio (OR 1.50, 95% CI 1.04–2.19, *p* = 0.03). These results suggest that the m¹A/A ratio may be considered as a potential biomarker for predicting - or aid in predicting - moderate to severe neurological outcome or death (CPC 2–5) after CA, while also reflecting molecular alterations associated with post-CA injury and outcome.

We next evaluated the incremental predictive value of the m¹A/A ratio to a baseline model including age, time between CA and ROSC, lactate, and NSE. The addition of m¹A/A ratio to the baseline model improved the quality of the baseline model, as attested by a reduction of the AIC from 254.11 to 185.4. This reduction was significant, as determined by the likelihood ratio test (*p* = 0.029). Reclassification analyses showed a NRI of 0.305 (*p* = 0.027), and an IDI of = 0.016 (*p* = 0.116). These results show that adding the m¹A/A ratio to a baseline clinical model may improve its capacity to predict neurological outcome or death.

### Assessment of the potential of m¹A/A ratio to predict survival

**Fig. 3:**
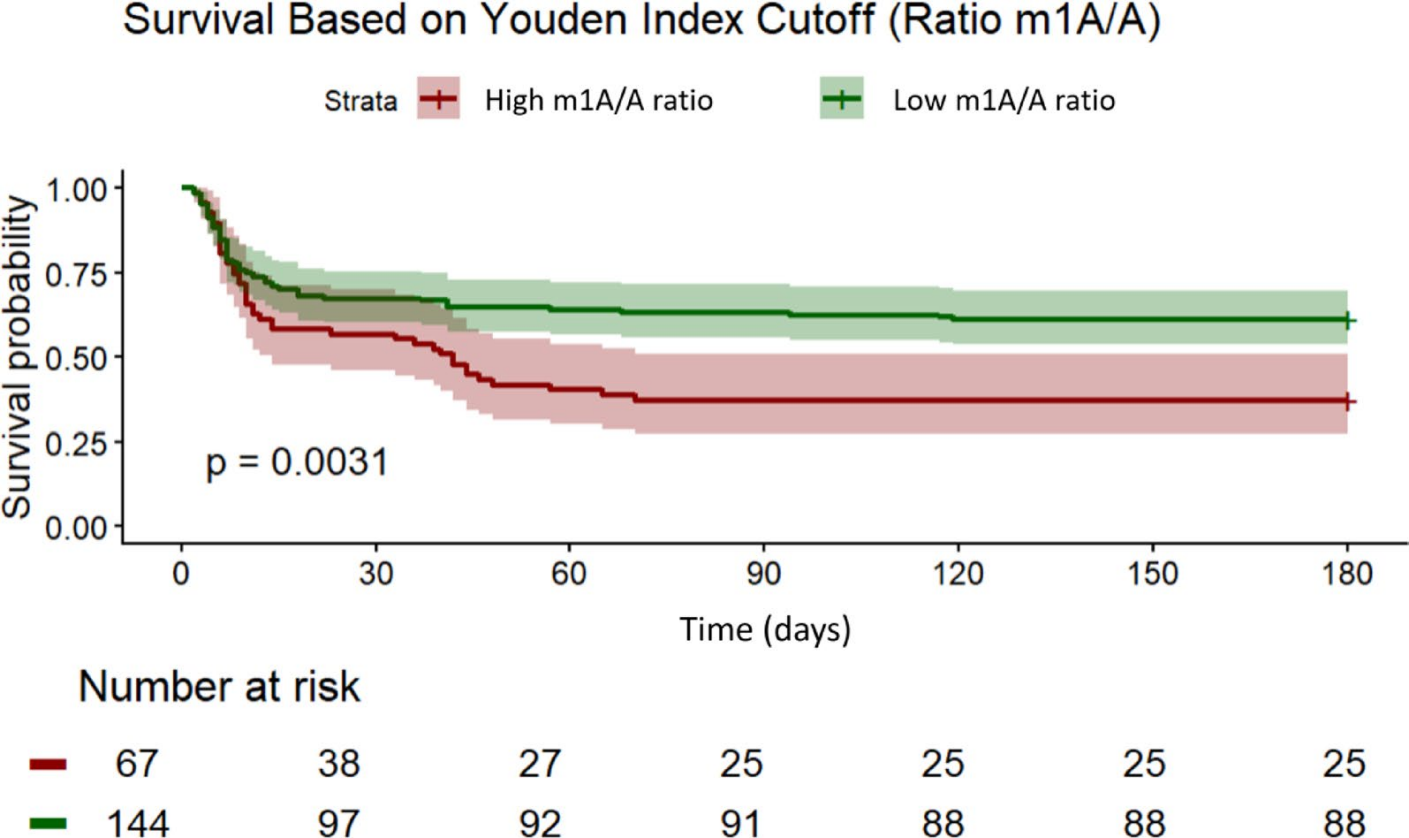
Survival analysis. Kaplan–Meier survival curves stratified by m¹A/A ratio in 211 CA patients are shown. Patients were divided into high (red, *n* = 67) and low (green, *n* = 144) m¹A/A ratio groups using the Youden Index as cutoff (Y = 0.20). The low m¹A/A group showed better survival compared to the high m¹A/A group group (log-rank test, *p* = 0.0031). Numbers of at risk patients are shown

Kaplan-Meier survival curves were generated to compare survival probabilities across different levels of the m¹A/A ratio. The Youden Index (Y = 0.20; best compromise between specificity and sensitivity) was used to determine the optimal cutoff value for the m¹A/A ratio, allowing for the stratification of patients into two distinct groups, patients with high (*n* = 67) and patients with low (*n* = 144) m¹A/A ratio. The survival curves showed a clear separation between both groups, with patients in the low m¹A/A ratio group showing better survival compared to those in the high m¹A/A ratio group (log-rank test, *p* = 0.0031; Fig. 3). This observation indicates that the m¹A/A ratio could serve as a potential predictive biomarker of survival in this patient population.

**Fig. 4:**
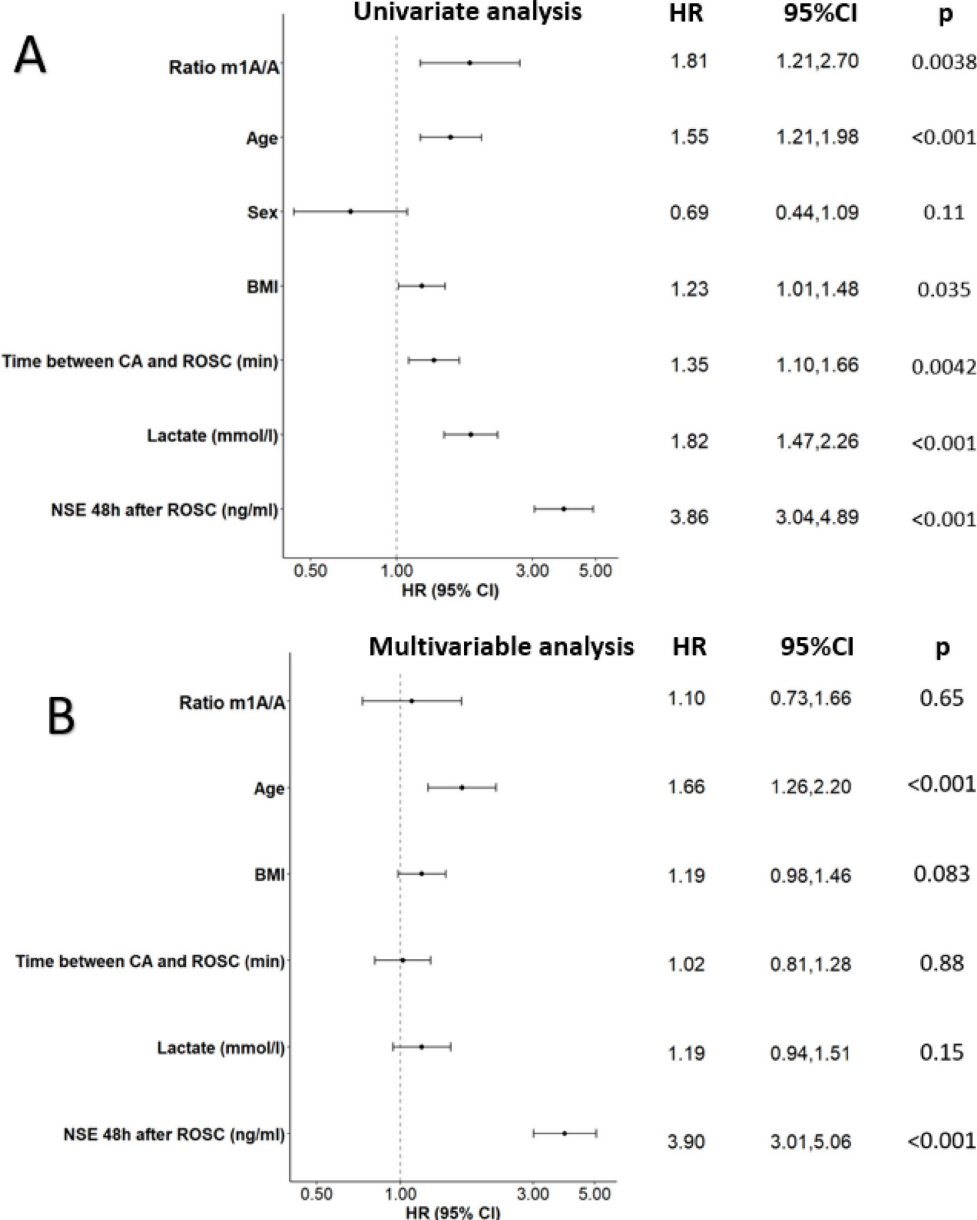
Univariate and multivariable Cox proportional hazards analysis for the prediction of death at 6 months in 211 CA patients. (**A**) In univariate analysis, higher m¹A/A ratio, age, BMI, time between CA and ROSC, lactate, and NSE at 48 h were significantly associated with increased risk of death. NSE demonstrated the strongest association.(**B**) In multivariable anaylsis, after adjustment for clinical covariates, m¹A/A ratio was not independently associated with 6-month mortality, while age and NSE remained significant predictors. NSE remained the strongest independent predictor. Cox proportional hazard regression analysis evaluating predictors of 6-month mortality after CA. Patients were dichotomized into high and low m¹A/A ratio groups based on the optimal cutoff determined by the Youden index (Y = 0.20), with the high group used as the reference category. Horizontal lines represent 95% confidence intervals for each hazard ratio (HR)

To further evaluate the capacity of the m¹A/A ratio to predict survival, we performed Cox proportional hazards regression analyses. Based on the optimal cutoff determined by the Youden index (Y = 0.20), patients were dichotomized into high and low m¹A/A ratio groups, with the high group used as the reference category. In the univariate model, a higher m¹A/A ratio was significantly associated with an increased risk of mortality (hazard ratio [HR] = 1.81, 95% CI: 1.21–2.70, *p* = 0.00376; Fig. 4A), indicating its potential as a predictor of survival and as a potential actor in the pathophysiology of CA injury and outcome. Other clinical parameters including age, BMI, time between CA and ROSC, lactate levels, and NSE at 48 h post-ROSC were also significantly associated with mortality. However, the association between m¹A/A ratio and survival lost statistical significance in the multivariable model after adjusting for confounding variables such as age, BMI, time between CA and ROSC, lactate, and NSE (Fig. 4B) (adjusted HR = 1.10, 95% CI: 0.73–1.66, *p* = 0.652). These results suggest that while the m¹A/A ratio is associated with survival in univariate analysis, its independent prognostic value may be limited when accounting for established clinical predictors. Spearman correlation analysis revealed only weak correlations between the m¹A/A ratio and covariates included in the basal model, such as age, BMI, time between CA and ROSC, lactate levels and NSE. This suggests that the loss of statistical significance in the multivariable analysis is unlikely to result from collinearity with these factors.

## Discussion

In this study, we provide the first evidence that m^1^A RNA modification is associated with neurological impairment and mortality following CA. This reveals a previously unexplored layer of CA biology, linking the epitranscriptome to neurological damage. The ratio between m¹A and adenosine (m¹A/A ratio) measured in blood 48 h post CA was found to be higher in patients developing neurological sequelae (ranging from moderate to severe sequelae) or who died within 6 months, as compared to survivors that fully recovered. Hence, CA patients with elevated m¹A/A ratio have a higher risk of important neurological sequelae and death. Adding m¹A/A ratio to a baseline clinical model improved its capacity to predict patient’s neurological outcome. This finding may have clinically relevant implications in post-CA management, since current multimodal prognostication strategies have some limitations. Improving prediction models may aid in early clinical decision-making that would benefit both patients and their relatives. In addition, such molecular insights could contribute to a better understanding of post-CA pathophysiology and the mechanisms underlying neurological injury and recovery.

Several RNA modifications were quantified in this study: N⁶-methyladenosine (m⁶A), N⁶−2′-O-methyladenosine (m⁶Am), 2′-O-methyladenosine (Am), β-pseudouridine (Ψ), and m1A. Since m1A and the m¹A/A ratio showed association with the primary endpoint of this study, i.e., neurological outcome at 6 months, we focused the paper on this particular modification, not excluding however that other RNA modifications may also be associated with outcome post-CA. Selection of m1A was hypothesis-driven, based on the knowledge that m¹A is enriched in mitochondrial RNAs and has been implicated in mitochondrial translation and energy metabolism [12], which are processes that are critically affected during global ischemia-reperfusion injury following cardiac arrest. Emerging evidence also links dysregulation of m¹A to cerebral ischemia and neurodegeneration [17, 18], supporting its potential relevance to neurological outcome. The measurement of m¹A in whole blood samples from CA patients has been assessed using a highly sensitive LC-MS approach following a protocol that we have previously developed and validated for other chemical modifications of adenosine residue [15]. While m^6^A is the most widely investigated RNA methylation [19], m¹A has received less attention. Its role in human disease, cardiovascular diseases and brain pathophysiology is only beginning to be unraveled [13, 18, 20]. However, this area holds great potential, as m¹A is emerging as a novel epitranscriptomic mark potentially involved in the pathophysiology of cardiovascular disorders and may serve as a valuable biomarker for prognosis and therapy, but also to understand the molecular insights of diseases [21]. We thus investigated m¹A in patients suffering from CA, in which the current biomarkers and multimodal prediction strategies often lack sensitivity and specificity. Interestingly, we observed that the m¹A/A ratio, used to circumvent inter-individual variability in total adenosine levels, was higher in patients with neurological impairment or death compared to survivors. This association with the neurological outcome suggests that the concept of epitranscriptomic altertions are part of the biological response to cardiac arrest. Reclassification analyses (NRI and IDI) and measures of predictive model improvement (AIC) support an incremental predictive value of the m¹A/A ratio to established indicators of patient’s neurological outcome, although the IDI did not reach statistical significance. In our survival analyses using Kaplan-Meier curves, the m¹A/A ratio was also associated with survival, reinforcing its potential as a biomarker of neurological injury and mortality. However, in multivariable Cox regression analysis, the association between the m¹A/A ratio and survival lost statistical significance after adjustment for covariates such as age, BMI, time between CA and ROSC, lactate, and NSE levels. Correlation analysis showed no strong associations between the m¹A/A ratio and the adjusted covariates (all |ρ| < 0.1), suggesting that the loss of significance was not due to collinearity. Thus, m¹A may capture a complementary biological signal that is not entirely independent from clinical predictors but still contributes to the overall pathophysiological landscape of post–CA injury. Compared with established biomarkers such as NSE or the emerging NfL biomarker, the discriminative performance of the m¹A/A ratio was modest, with considerable overlap between groups. NfL measures were not available in our cohort, precluding direct comparison. The aim of the present work was not to position m¹A/A as a standalone alternative to current biomarkers, but rather to explore whether epitranscriptomic modifications may provide incremental prognostic information and that it may also provide additional biological insights. Despite its modest effect size, the inclusion of m¹A/A improved model performance through reductions in AIC and improvements in reclassification metrics, suggesting possible additive value.

The mechanisms underlying the regulation of m¹A RNA modification after CA, as well as in other situations, remain to be elucidated. However, some hypotheses can be proposed. m1A is found in many mitochondrial transfer and ribosomal RNAs, thereby influencing mitochondrial function such as mitochondrial RNA stability and translation efficiency [22]. In the context of CA, global ischemia-reperfusion injury induces mitochondrial dysfunction, impaired oxidative phosphorylation, increased reactive oxygen species production and metabolic failure. Given the established role of m¹A in regulating mitochondrial RNA translation, altered m¹A/A ratio may reflect disrupted mitochondrial RNA metabolism in response to ischemic stress. Therefore, m^1^A may contribute to defective mitochondria and energetic dysregulations. Such alterations may amplify neuronal and systemic injury, linking defective m¹A regulation to moderate to severe neurological outcome and survival. An alternative explanation for the increased m¹A/A ratio is the release of chemically modified RNAs from injured tissues. Global ischemia-reperfusion injury causes extensive cellular damage in the brain, leading to the disruption of the blood-brain barrier, which can subsequently allow the leakage of RNA species from the brain into the bloodstream. Thus, high levels of circulating m¹A could reflect the level of brain tissue injury and disruption of the blood-brain barrier. Understanding the mechanisms driving m¹A alterations after CA will be essential to determine whether the differences in m1A levels between patient groups observed in the present study are reflecting a byproduct of brain tissue damage or play an active role in the pathophysiology of CA.

Also, the altered m¹A/A ratio associated with the neurological injury have implications beyond prognosis, pointing to a potential role for epitranscriptomic regulation in post-cardiac arrest disease mechanisms. Dynamic regulation of m¹A methylation occurs through specific writer and eraser enzymes, including TRMT6/TRMT61A complex for cytosolic RNAs and TRMT61B and ALKBH1 for mitochondrial RNAs [12], with established connections to mitochondrial RNA metabolism and cellular stress responses. While the present data do not justify therapeutic approaches targeting m¹A pathways, they suggest the hypothesis that RNA methylation manipulation might influence outcomes after global ischemia-reperfusion injury. Whether m¹A-related mechanisms represent actionable therapeutic targets remains to be explored through future experimental investigations.

This study has some limitations. First, the focus on m^1^A in the present study was not consecutive to post-hoc testing for multiple comparisons but was hypothesis-driven and linked to the strongest association with the primary end-point of the study among the five RNA modifications analysed by LC-MS. Second, the relatively small sample size and monocentric feature of the study cohort might reduce the statistical power to detect smaller effects. Validation in larger, independent cohorts will be necessary to confirm present findings, strengthen our conclusions, and better capture biological variability. Third, all analyses were performed on samples collected 48 h after CA. In the North Pole cohort, 48 h represented the earliest standardized sampling time point available. While this time point was chosen based on clinical relevance and previous studies addressing the prognostic value of other RNA-based biomarkers [9, 16, 23], it is possible that changes in the m¹A/A ratio could occur earlier (within 24 h after CA), which could advance the prognostic capacity of NSE for instance [24]. Studying m1A methylation at early stages after CA may also provide additional insights into the dynamics of RNA modifications and their potential functional role in post-CA injury and outcome. In addition, CA location (in-hospital or out-of-hospital), first rhythm or witnessed status are known to be associated with outcome post-CA, these variables were not included in the prediction model to avoid over-fitting due to low sample size and overlap with ischemic time and indicators of neurological damage. Fourth, m1A and A levels were measured in whole blood cell samples which, although being an easily accessible and non-invasive matrix, may not fully represent the events occurring in the brain after CA. It may be valuable in future studies to investigate m¹A levels in other biological fluids (if detectable) such as plasma, serum, or cerebrospinal fluid. Studying m^1^A in post-mortem brain tissue, isolated cell populations, or brain organoids would allow exploring potential cell-specific signals and functional aspects. Another important point is that, although the RNA extraction and LC-MS workflow used in this study is not directly applicable to routine clinical practice, it serves as a first proof-of-concept to demonstrate the biological relevance of epitranscriptomic alterations after cardiac arrest. Translation into clinical prognostication would require the development of simplified, standardized, and potentially automated assays. Finally, our outcome categorization (CPC 1 vs. CPC 2–5) differs from conventional definitions of CPC 1–2 as good outcome and CPC 3–5 as poor outcome. This approach was defined to distinguish fully preserved neurological function from any measurable impairment.

## Conclusions

Our study shows for the first time that the m¹A/A RNA modification ratio in whole blood is associated with outcome after CA. Although at an early stage, these findings suggest that m¹A/A may provide complementary prognostic information as well as novel insights into the molecular mechanisms underlying post-CA injury. While m¹A/A ratio may not be used as a standalone biomarker, it may provide incremental predictive value to current multimodal prognostication strategies. Further studies in larger patient cohorts are needed to validate these findings and confirm the potential prognostic and biological relevance of m¹A RNA modification. Further biological studies are required to understand also the mechanisms underlying epitranscriptomic regulation after CA.

## Data Availability

The data generated and analyzed in this study is available upon request to authors.
